# Ergot alkaloid control in biotechnological processes and pharmaceuticals (a mini review)

**DOI:** 10.3389/ftox.2024.1463758

**Published:** 2024-10-08

**Authors:** A. Volnin, A. Parshikov, N. Tsybulko, P. Mizina, N. Sidelnikov

**Affiliations:** ^1^ Laboratory of Biotechnology, All-Russian Research Institute of Medicinal and Aromatic Plants (VILAR), Moscow, Russia; ^2^ Center of Chemistry and Pharmaceutical Technology, All-Russian Research Institute of Medicinal and Aromatic Plants (VILAR), Moscow, Russia; ^3^ All-Russian Research Institute of Medicinal and Aromatic Plants (VILAR), Moscow, Russia

**Keywords:** ergot alkaloids, ergoalkaloids, qualitative analysis, quantitative analysis, express methods, high-precision methods, HPLC, process analytical technology

## Abstract

The control of ergot alkaloids in biotechnological processes is important in the context of obtaining new strain producers and studying the mechanisms of the biosynthesis, accumulation and secretion of alkaloids and the manufacturing of alkaloids. In pharmaceuticals, it is important to analyze the purity of raw materials, especially those capable of racemization, quality control of dosage forms and bulk drugs, stability during storage, etc. This review describes the methods used for qualitative and quantitative chemical analysis of ergot alkaloids in tablets and pharmaceutic forms, liquid cultural media and mycelia from submerged cultures of ergot and other organisms producing ergoalkaloid, sclerotias of industrial *Claviceps spp*. parasitic strains. We reviewed analytical approaches for the determination of ergopeptines (including their dihydro- and bromine derivatives) and semisynthetic ergot-derived medicines such as cabergoline, necergoline and pergolide, including precursors for their synthesis. Over the last few decades, strategies and approaches for the analysis of ergoalkaloids for medical use have changed, but the general principles and objectives have remained the same as before. These changes are related to the development of new genetically improved strains producing ergoalkaloids and the development of technologies for the online control of biotechnological processes and pharmaceutical manufacturing (“process analytical technologies,” PAT). Overall, the industry is moving toward “smart manufacturing.” The development of approaches to production cost estimation and product quality management, manufacturing management, increasing profitability and reducing the negative impact on personnel and the environment are integral components of sustainable development. Analytical approaches for the analysis of ergot alkaloids in pharmaceutical raw materials should have high enough specificity for the separation of dihydro derivatives, enantiomers and R-S epimers of alkaloids, but low values of the quantitative detection limit are less frequently needed. In terms of methodology, detection methods based on mass spectrometry have become more developed and widespread, but NMR analysis remains in demand because of its high accuracy and specificity. Both rapid methods and liquid chromatography remain in demand in routine practice, with rapid analysis evolving toward higher accuracy owing to improved analytical performance and new equipment. New composite electrochemical sensors (including disposable sensors) have demonstrated potential for real-time process control.

## Introduction

Ergot *Claviceps purpurea* (Fries) Tulasne is of critical economic importance because it is a producer of many biologically active compounds (alkaloids) for the pharmaceutical industry, a unique model of the parasite-host system, and a mycotoxin-associated pathogen that causes significant economic damage to agriculture around the world (Volnin and Savin, 2022). To meet the global demand for ergot alkaloids, 8 tons of ergopeptines and up to 10–15 tons of D-lysergic acid are produced each year. Approximately 60% of these strains are produced by the submerged fermentation of specially developed strains of *C. purpurea*, whereas the rest are obtained from field cultivation (Wong et al., 2022).

The analysis of ergot alkaloids is an important topic in safety programs for food, animal feed and other agricultural products. The European Food Safety Authority (EFSA) conducts monitoring to assess its presence in food and animal feed (Holderied et al., 2019). In the Russian Federation, the Federal Service for Veterinary and Phytosanitary Surveillance (Rosselkhoznadzor of the Russian Federation) is responsible for regulatory procedures and monitoring ergot in agricultural products (Pankin et al., 2023).

Rapid methods used for the determination of ergot alkaloids in agricultural products include spectrophotometric determination of alkaloid sum (Sheshegova et al., 2019, 2021), hydrazinolysis with determination of the total precursor content (Kuner et al., 2021), thin-layer chromatography (TLC) (Sheshegova et al., 2019, 2021), high-performance thin-layer chromatography with fluorescence detection (HPTLC-FLD) (Oellig, 2017), planar solid-phase extraction with fluorescence detection (Oellig and Melde, 2016; Chung, 2021), enzyme-linked immunoassay (ELISA) (Gross et al., 2018; Kodisch et al., 2020; Boško et al., 2024), immunoassays with magnetic beads and amperometric detection (Soraya et al., 2023; Silva et al., 2023), and capillary electrophoresis (Felici et al., 2015).

High-precision methods for the determination of ergoalkaloids in agricultural products include liquid chromatography with UV or fluorometric detection (LC-UV, LC-FLD) (Chung, 2021; Crews, 2015); mass spectrometry-based methods (Bryła et al., 2015; Carbonell-Rozas et al., 2022), including liquid chromatography with tandem mass spectrometric detection (LC‒MS/MS) (Carbonell-Rozas et al., 2021a,b; Arroyo-Manzanares et al., 2021; Veršilovskis et al., 2020; Poapolathepet al., 2021); and gas chromatography‒mass spectrometry (Crews, 2015). Although LC‒MS/MS has dominated in practice in recent years, LC-FLD is still more common (Holderied et al., 2019).

Methods for the determination of ergoalkaloids in tablets should be rapid and specific, with low values of the quantitative detection limit being less frequently required (Crews, 2015).

The control of ergot alkaloids in biotechnological processes is important in the context of obtaining new strain producers and studying the mechanisms of the biosynthesis, accumulation and secretion of alkaloids and the manufacturing of alkaloids. In pharmaceuticals, it is important to analyze the purity of raw materials, especially those capable of racemization, quality control of dosage forms and bulk drugs, stability during storage, etc.

This paper characterizes methods of qualitative and quantitative chemical analysis of ergoalkaloids in tablets and pharmaceutic forms, sclerotia of industrial parasitic strains of *Claviceps* spp. and liquid cultural media or mycelia of ergot submerged cultures and other microorganisms used for ergoalkaloid production. Practical examples of the use of rapid methods, process analytical technology and high-precision analysis, including mass spectrometry-based methods, NMR and crystallography, are given. Analytical approaches for quality control of medicinal raw materials are considered, taking into account factors such as the epimerization of alkaloids and others.

## Isomerization, epimerization and dihydro derivatives of ergoalkaloids

Molecules of ergoalkaloids can epimerize relative to the center of symmetry at the C8 position (R- and S- epimers), and these forms can interconvert under the action of UV, temperature, pH and chemical reagents (Chung, 2021; Silva et al., 2023; Crews, 2015). The epimerization of alkaloids is important because of the different biological activities and toxicity of the R and S forms (however, recent studies have suggested the opposite (Cherewyk et al., 2020; 2024)), the possibility of epimerization during sample preparation and storage (Chung, 2021; Silva et al., 2023; Crews, 2015), and the influence of epimerization on the technological processes of obtaining drug raw materials (presence of impurities and quantitative yield of target alkaloids) (Komarova and Tolkachev, 2001a; Komarova and Tolkachev, 2001b; Zvonkova et al., 2000).

Ergotamine and ergosine are very stable ergot alkaloids, and neither their concentrations, nor their respective R/S ratios, are significantly influenced by heat, protic solvents or UV light (Schummer et al., 2020). In contrast, for ergocristine, ergokryptine, ergocornine and ergometrine, heat can decrease the concentrations of these alkaloids, and heat, protic solvents and UV light influence the R/S ratio toward the S-form, although the respective influences on the epimerization of these compounds are variable (Schummer et al., 2020).

Liquid chromatography-fluorescence (Holderied et al., 2019) or UV- (Zvonkova et al., 2000) detection, capillary electrophoresis, liquid chromatography‒mass spectrometry (Cherewyk et al., 2024), and ion mobility mass spectrometry (Carbonell-Rozas et al., 2022) can be used to quantify both R- and S-epimers of ergot alkaloids (Cherewyk et al., 2024).

The synthesis of pharmaceutical substances such as modified ergoalkaloids (in particular bromine derivatives) is markedly complicated by their lability and susceptibility to epimerization. The epimerization process is in equilibrium, proceeds with heating, and is catalyzed by acids and bases. The formation of epimers at C8 results in the loss of the target compound with a noncrystallizable reaction mixture and with the head fractions during chromatographic purification involved in parent substance synthesis (Zvonkova et al., 2000). Methods for reverse epimerization have been developed and presented (Zvonkova et al., 2000).

For the manufacturing of semisynthetic ergoalkaloids, such as cabergoline, nicergoline and pergolide, an important aspect is the presence of a branch point associated with the isomerization of paspalic acid to lysergic acid [Liu and Jia, 2017; Himmelsbach et al., 2014). Since this process does not tend toward chemical balance, the racemized products were carefully purified. This continuous improvement of strains to increase productivity and optimize fermentation parameters related to biotechnological processes implies the need to develop methods for analyzing samples at different stages of alkaloid manufacturing (Himmelsbach et al., 2014).

An intermediate process for the semisynthesis of cabergoline and pergolide is hydrogenation [Liu and Jia, 2017). In addition, dihydroalkaloids are also the active substances of some drugs (e.g., dihydroergotamine). (Schiff, 2006). Analytical approaches for the analysis of ergot alkaloid raw pharmaceutical materials should have high enough specificity for the separation of dihydro derivatives, enantiomers and R-S epimers of alkaloids.

## Methods of analyses

### Spectrofluorimetry and spectrophotometry

Many ergoat alkaloids, including the most common, are naturally fluorescent. The method of fluorescence spectroscopy (spectrofluorimetry) possesses high sensitivity, being capable of detecting about 1 ng of lysergic acid derivatives (Komarova and Tolkachev 2001b).

The spectrophotometric technique used for the quantitative determination of ergot alkaloids offers high sensitivity. However, the method is insufficiently selective (Komarova and Tolkachev 2001b). Nonselective spectrophotometric determination of total alkaloid content with van Urk reagent is used for rapid analysis of sclerotia of ergot industrial parasitic strains. The disadvantage of this method is its sensitivity to all substances containing indole groups, including tryptophan (Volnin et al., 2022).

### Thin-layer chromatography

Thin layer chromatography (TLC) is a qualitative, selective method but can be used both separately and in combination with spectrophotometric, spectrofluorimetric quantitative (Komarova and Tolkachev 2001b) or densitometric semiquantitative analysis (Komarova and Tolkachev 2001b; Volnin et al., 2022,^2023^).

Thin-layer chromatography can be used for studying the stability and degradation pathways of related drugs during storage and determining the content of impurities in parent substances (Komarova and Tolkachev 2001b). The disadvantages of this method are different selectivities for different groups of alkaloids, different sensitivities to eluent composition and different conditions of analysis (Volnin et al., 2022).

### Voltammetry

Electrochemistry has many benefits, making it an appealing choice for clinical, food, pharmaceutical and environmental studies. Electrochemistry has provided analytical techniques characterized by instrumental moderate cost, simplicity, and portability (Mohammadi et al., 2019).

### Capillary electrophoresis

Capillary electrophoresis has been used for the determination of ergoalkaloids and some epimers. Good separation was achieved but for a limited number of alkaloids (Crews, 2015).

### HPLC

HPLC is widely used for both the qualitative and quantitative analysis of ergot alkaloids, for the characterization of raw materials (ergot sclerotias and saprophytic cultures), and for the investigation of technological products and medicinal preparations containing ergot alkaloids (Komarova and Tolkachev, 2001b).

Reversed-phase chromatography is always used for the separation of ergoalkaloids. Most methods use solvent systems of methanol–water or acetonitrile–water mixtures with ammonium hydroxide, ammonium carbonate, ammonium carbamate or triethylamine to provide alkaline pH conditions. Separation can be achieved with both isocratic and gradient mobile phases. Alkaline mobile phases are preferred to maintain the stability of both epimers, avoid protonation and improve separation. (Crews, 2015).

Both ultraviolet (UV) and fluorescence (FLD) have been coupled with LC for ergot alkaloids analysing. Since many ergot alkaloids, including the most common, are naturally fluorescent (Komarova and Tolkachev, 2001b) and FLD offers better sensitivity and specificity than UV, detection with UV has become less common (Chung, 2021; Crews, 2015; Komarova and Tolkachev, 2001b). With LC separation, ergopeptines and ergopeptinines can be measured with an ultraviolet (LC-UV) detector set to a wavelength maxima of 310 nm for ergopeptines and ergopeptinines and at 280 nm for dihydroergopeptines, although other wavelengths have been included (Crews, 2015; Blaney et al., 2009; Komarova and Tolkachev, 2001b).

### Mass spectrometry

Mass spectrometry is used for identifying ergot alkaloids, determining their structures, and characterizing the peptide fragments of molecules (Komarova and Tolkachev, 2001b). Determination by high-performance liquid chromatography (HPLC) coupled with tandem mass spectrometry (LC‒MS/MS) has become a standard approach for trace quantification and identification (Crews, 2015).

### NMR

NMR spectroscopy is one of the main methods used for identifying ergot alkaloids; studying the stereoconfiguration, structure, and lability of molecules; and solving problems related to changes in the configuration and structure of these compounds. Using this method, it is possible to determine the ratio of alkaloids in a mixture while simultaneously confirming the component structures, which facilitates the solution of many technological problems (Komarova and Tolkachev, 2001b).

## Operational control of bioprocesses

A current trend in the pharmaceutical and biotechnology industry is noninvasive real-time operational control of technological processes (“process analytical technology”, PAT) (Gillespie et al., 2022).

This approach can potentially be realized by combining spectroscopic measurements (UV/visible spectroscopy, IR spectroscopy, fluorescence spectroscopy and Raman spectroscopy) with multivariate data analysis (Claßen et al., 2017; Esmonde-White et al., 2017; Schlembach et al., 2021). Electroanalytical methods are characterized by instrumental simplicity, moderate cost, reasonable accuracy, precision, and speed (Hasanpour et al., 2017). Currently, improving novel sensing materials such as graphene, nanoparticles, and carbon nanotubes that are capable of enhancing the analytical properties of electrode surfaces is vital. Disposable sensors based on screen-printed electrodes have led to innovative possibilities for analytic quantitation (Mohammadi et al., 2019).

With advances in production processes, especially in the biopharmaceutical and nutraceutical industries, monitoring and control of bioprocesses such as fermentation with GMO organisms and downstream processing has become increasingly complex, and the inadequacies of classical and some modern control system techniques are becoming apparent. Therefore, with increasing research complexity, nonlinearity, and digitization in the process, there has been a critical need for advanced process control that is more effective, and easier process intensification and product yield (both by quality and quantity) can be achieved (Mitra and Murthy, 2022).

Bioprocess control involves more than just automation and includes aspects such as system architecture, software applications, hardware, and interfaces, all of which are optimized and compiled per demand. This needs to be accomplished while maintaining process requirements, production costs, the market value of products, regulatory constraints, and data acquisition requirements (Rathore et al., 2021). Expansion of the Process Analytical Technology toolbox could lead to “smart manufacturing” (Isoko et al., 2024). However, several unique and dominant features of biobased processes call for methods and methodologies that differ from those that have been successfully applied to large-scale continuous chemical processes or discrete-parts manufacturing processes (Bähner et al., 2021).

## Ergot alkaloid production and control in biotechnological processes

The literature indicates possible problems with the identification of alkaloids by HPLC when analyzing wild ergot. Blaney et al. reported several problems with the identification of alkaloids from the ergotoxin group (in *C. purperea* sclerotias from grain samples) (Blaney et al., 2009). The same problems may arise in the determination of ergotoxin alkaloids in industrial parasitic strains. More careful selection of conditions for chromatographic separation or additional control by NMR may solve this problem (Komarova and Tolkachev, 2001b). NMR spectra of the ergototoxin alkaloids (ergocryptines, ergocristine, ergocornine) present in the sclerotia of industrial parasitic ergot strains were obtained. These data may be useful for identifying the α and β forms of ergocryptine and estimating the impurity content in the pharmaceutical manufacturing of alkaloids (Komarova and Tolkachev, 2001b).

NMR and crystallographic methods have been used for the characterization of intermediates in full chemical synthesis methods of ergot alkaloids (Jastrzębski et al., 2022; Brauer et al., 2024). Ergot alkaloids obtained from platforms based on *Saccharomyces cerevisiae* (Nielsen et al., 2014) and *Aspergillus nidulans* (Qiao et al., 2022) were characterized via NMR.

Capillary zone electrophoresis was used for analysis of paspalic, lysergic and iso-lysergic acids in combination with UV and quadrupole mass spectrometric detection [Himmelsbach et al., 2014).

In the study of a submerged culture of a *C. paspali* strain with an inactivated gene cluster for the synthesis of indolediterpenes (which are toxic byproducts in the production of ergoalkaloids), a complex problem was solved by combining HPLC MS/MS for the determination of paspaline, paspalinine, paxiline and paspalitremes and two HPLC-UV modes for ergot alkaloids (two elution modes and two wavelengths of 310 and 230 nm). Since lysergic acid and paspalic acid co-eluted under standard conditions, the separation of these compounds was achieved via a different HPLC method with a new elution mode and UV detection at λ = 230 nm (Kozák et al., 2018).

HPLC with fluorescence detection and LC‒MS were used for the determination of alkaloids synthesized by modified strains of *Metarhizium brunneum* [Davis et al., 2020, 2023) and *C. paspali* (Hu et al., 2023); lysergic acid; ergometrine produced by *C. paspali* (Qiao et al., 2022); and clavine alkaloids and dihydroderivatives produced in a heterologous system based on *A. nidulans* (Yao et al., 2022). High-performance liquid chromatography‒tandem mass spectrometry (HPLC‒MS/MS) was used for the determination of lysergic acid produced by transgenic yeast (Lim et al., 2023; Wong et al., 2022).

Reversed-phase HPLC with elution in gradient mode with formic acid solutions in acetonitrile and water can also be used for the analysis of ergoalkaloids [Doi et al., 2022; Ma et al., 2022), including mass spectrometric detection (Ma et al., 2022). In addition, aqueous ammonium formate solution and acetonitrile can be used as solvents (Chen et al., 2019). HPLC in gradient mode with elution of aqueous solutions of acetic acid and acetonitrile was used for the determination of ergopeptines produced in submerged culture by genetically modified ergot strains (Králová et al., 2021).

The van Urk reaction has been used for the determination of ergoalkaloids produced *in vitro* by mutant strains of *Penicillium citrinum* [Shahid et al., 2020) and *C. purpurea* (Bobyleva and Savin, 2021; Bobyleva and Tsybulko, 2021). Thin layer chromatography is used for rapid and selective confirmation of alkaloid synthesis for saprophytic strain selection and rapid identification of parasitic strains through simple chemotaxonomic markers (Volnin et al., 2022, 2023).

Fluorescence spectroscopy is a convenient method for real-time on-line monitoring of microbial fermentation processes and allows the measurement of substrate and product concentrations and the evaluation of process status (Faassen and Hitzmann, 2015; Alemneh et al., 2022,^2023^). In particular, the application of two-dimensional fluorescence spectroscopy and chemometric models (biomass, protein, and alkaloid concentrations) for real-time analysis (online monitoring) of bioprocesses in a bioreactor during cultivation of *C. purpurea* via a BioView^®^ sensor (DELTA Light and Optics, Denmark) was described (Boehl et al., 2003).

## Pharmaceutical applications

Control of ergot alkaloids is necessary for the analysis of tablet and injectable dosage forms as well as bulk drugs. In pharmaceuticals, it is important to analyze the purity of raw materials; the contents of active ingredients, impurities and additives; the quality control of finished dosage forms; and the stability during storage. Quality-control procedures need selective, accurate, economical, simple, rapid stability-indicating methods to solve analytical problems (Farid and Abdelwahab 2019; Jyothirmayee and Raju, 2014; Önal et al., 2007). Finished dosage forms are multicomponent, and their analysis requires sensitive and easy separation and simultaneous determination of components without interference with the additives and excipients normally used in tablet formulations (Al-Akraa and Kabaweh, 2015). The methods used are summarized in Table 1.

**TABLE 1:** Determination of ergot alkaloids in pharmaceutical dosage form and bulk drugs.

No	Alkaloid	Method	Characteristics	References
Express methods				
1	Bromocriptine mesylate	Spectrofluorimetry	Sensitive, precise, quick, and affordable	Abdulrazik et al. (2023)
2	Cabergoline	Spectrofluorimetry	Simple, economic, and selective	Rizk et al. (2022)
3	Cabergoline	Spectrophotometry	Simple, selective and accurate	Rambabu et al. (2012)
4	Nicergoline and its degradation products	Spectrophotometry	The first method was based on measurement of the first derivative of ratio spectra amplitude of nicergoline at 291 nm	Ahmad et al. (2002)
5	Nicergoline and its degradation products	HPTLC	Separation of nicergoline from its degradation product followed by densitometric measurement of the spots at 287 nm	Ahmad et al. (2002)
6	Cabergoline	Thin layer chromatographic (TLC) with fluorescence detection	Stability-indicating method	Rizk et al. (2022)
7	Ergotamine	High-performance thin-layer chromatography (HPTLC)	Determined simultaneously with caffeine and metamizole	Crews (2015)
8	Cabergoline and its degradation products	HPTLC	Quick determination of quantificate and stability	Farid and Abdelwahab (2019)
9	Bromocriptine	Voltammetry	Verification the uniformity content of bromocriptine in commercial tablets	Radi et al. (2005)
10	Ergotamine tartrate	Capillary electrophoresis	Long analysis time	Sultan et al., 2013; Crews, 2015
High-precision methods				
11	Cabergoline	HPLC	Simple, specific	Farid and Abdelwahab 2019; Jyothirmayee and Raju, 2014
12	Cabergoline	HPLC	Mobile phase containing Acetonitrile, phosphoric acid, triethylamine	Önal et al. (2007)
13	Nicergoline	HPLC	Mobile phase containing methanol, acetonitrile and ortho phosphoric acid	Kumar and Nadh (2011)
14	Nicergoline	HPLC	Mobile phase of methanol-water-glacial acetic acid	Ahmad et al. (2002)
15	Pergolide	Ion-pair chromatography	Stability indicating	Kotzagiorgis et al. (2007)
16	Pergolide	HPLC	Stability indicating	Shank and Ofner (2010)
17	Dihydroergotamine	Ion-pair UPLC	Quantify the related substances for injection drug product	Basappa et al. (2024)
18	Ergotamine tartrate	HPLC	Isocratic mode with methanol/formic acid solvents and UV detection	Ashour and Omar (2013)
19	Bromocriptine mesylate	HPLC	Elution by methanol solution of formic acid	Ashour and Kattan (2013)
20	Bromocriptine mesylate	HPLC	Elution by methanol solution of sodium acetate	Pukngam and Burana-osot (2013)
21	Dihydroergocristine	HPLC	Fluorescence detection and elution by potassium dihydrogen phosphate buffer -acetonitrile	Dousa and Dubovská (2010)
22	Dihydroergocristine mesylate mixed with clopamide and reserpine	HPLC	The mobile phase consists of a mixture of ammonium acetate, acetonitrile and methanol	Al-Akraa and Kabaweh (2015)

Quality control procedures can have certain problems depending on the substance being analyzed. For example, cabergoline exhibits poor UV absorbance and fluorescence, as well as little thermal stability and low volatility; as a result, some analytical methods, such as high-performance liquid chromatography with UV or fluorescence detectors and gas chromatographic techniques, are not always convenient for sensitive determination (Hasanpour et al., 2017).

A screen-printed electrode (SPE) modified with a La2O3/Co3O4 nanocomposite (La2O3/Co3O4/SPE) has been developed and employed for sensitive and selective quantification of the ergot derivative drug cabergoline with exceptional accuracy and precision in a phosphate buffer solution via different pulse voltammetry techniques (Mohammadi et al., 2019). A novel maghemite nanoparticle carbon paste-modified electrode was developed for the determination of cabergoline. The modified electrode has an outstanding catalytic effect on the oxidation current of cabergoline, and the mechanism was studied via cyclic voltammetry. The proposed method was examined as a selective, simple and precise method for voltammetric determination of cabergoline in pharmaceutical samples (Hasanpour et al., 2017).

NMR analysis was used for characterization of impurities in the dihydroergotamine injection drug product (Basappa et al., 2024). NMR studies of ergometrine, methylergometrine and their maleate salts were carried out to determine their conformational parameters. In addition, the stereochemistry and intermolecular interactions in the solid-state of the two maleate salts were investigated via monocrystal X-ray diffraction (Meneghetti et al., 2020).

## Conclusion

Phytosanitary and toxicological analyses have certain specificities and may differ from analytical control procedures for biomedical ergot alkaloids in terms of several parameters. For example, toxicological methods require low detection limits and high sensitivity, low specificity of expression methods (e.g., immunoenzyme assays), additional sample preparation and purification procedures, a large variety of alkaloids with limited availability of standard solutions, and the presence of other indole-containing mycotoxins in samples.

The content of ergoalkaloids in sclerotias of industrial parasitic strains of *Claviceps spp*. and liquid cultural medias or mycelia of submerged cultures of ergot and other microorganisms that produce ergot alkaloids is generally greater than that of “wild” strains in agricultural and food toxicology. However, analytical approaches for the analysis of pharmaceutical raw materials should have sufficiently high specificity and selectivity for separating dihydro derivatives, enantiomers and epimers of ergoalkaloids. This directly affects the quality of the obtained raw pharmaceutical substances (the presence of impurities and the quantitative yield of the target alkaloid).

## References

[B1] Abdulrazik S. G. Attia T. Z. Derayea S. M. (2023). The first spectrofluorimetric protocol for sensitive quantitative analysis of bromocriptine in its pure and pharmaceutical forms: evaluation of the greenness of the method. RSC Adv. 13, 35733–35740. 10.1039/D3RA06626F 38077977 PMC10704114

[B2] Ahmad A. K. Kawy M. A. Nebsen M. (2002). First derivative ratio spectrophotometric, HPTLC-densitometric, and HPLC determination of nicergoline in presence of its hydrolysis-induced degradation product. J. Pharm. Biomed. Anal. 30 (3), 479–489. 10.1016/s0731-7085(02)00132-2 12367672

[B3] Al-Akraa H. Kabaweh M. (2015). Development and validation of rp-hplc method for simultaneous determination of clopamide, dihydroergocristine mesylate and reserpine in tablets. Int. J. Pharm. Pharm. Sci. 7 (7), 148–152.

[B4] Alemneh S. T. Babor M. Zettel V. von Wrochem A. Hitzmann B. (2023). Online monitoring of the growth of probiotic bacteria and metabolites in the fermentation of a teff substrate using model-based calibration of 2D fluorescence spectra. Microorganisms 11 (4), 1032. 10.3390/microorganisms11041032 37110455 PMC10143008

[B5] Alemneh S. T. Emire S. A. Jekle M. Paquet-Durand O. von Wrochem A. Hitzmann B. (2022). Application of two-dimensional fluorescence spectroscopy for the on-line monitoring of teff-based substrate fermentation inoculated with certain probiotic bacteria. Foods 11 (8), 1171. 10.3390/foods11081171 35454758 PMC9025233

[B6] Arroyo-Manzanares N. Rodríguez-Estévez V. García-Campaña A. M. Castellón-Rendón E. Gámiz-Gracia L. (2021). Determination of principal ergot alkaloids in swine feeding. J. Sci. Food Agric. 101 (12), 5214–5224. 10.1002/jsfa.11169 33609041

[B7] Ashour S. Kattan N. (2013). New sensitive method for determination of bromocriptine in tablets by high performance liquid chromatography. Res. J. Aleppo Univ. 87, 1–10.

[B8] Ashour S. Omar S. (2013). Sensitive method for the quantitative determination of ergotamine in tablet dosage form by high-performance liquid chromatography using bromocriptine as internal standard. Int. Res. J. Pure Appl. Chem. 3, 286–298. 10.9734/IRJPAC/2014/4528 PMC3708273 23847459

[B9] Bähner F. D. Prado-Rubio O. A. Huusom J. K. (2021). Challenges in optimization and control of biobased process systems: an industrial-academic perspective. Industrial Eng. Chem. Res. 60 (42), 14985–15003. 10.1021/acs.iecr.1c01792

[B10] Basappa P. Shankar U. Dama V. R. (2024). Isolation and characterization of major degradants in dihydroergotamine injection. Acta Chromatogr. 36 (2), 86–98. 10.1556/1326.2022.01095

[B11] Blaney B. J. Molloy J. B. Brock I. J. (2009). Alkaloids in Australian rye ergot (*Claviceps purpurea*) sclerotia: implications for food and stockfeed regulations. Anim. Prod. Sci. 49, 975–982. 10.1071/AN09030

[B12] Bobyleva R. I. Savin P. S. (2021). Analysis of morphological, physiological and biochemical features of saprophytic culture of *Claviceps purpurea* (Fries) Tulasne bkmf-2641d strain. Problems Biol. Med. Pharm. Chem. 24 (12), 57–62. 10.29296/25877313-2021-12-09

[B13] Bobyleva R. I. Tsybulko N. S. (2021). Study of growth and biosynthetic features of saprophytic claviceps purpurea lines in submerged culture. Problems Biol. Med. Pharm. Chem. 24 (7), 45–49. 10.29296/25877313-2021-07-07

[B14] Boehl D. Solle D. Hitzmann B. Scheper T. (2003). Chemometric modelling with two-dimensional fluorescence data for *Claviceps purpurea* bioprocess characterization. J. Biotechnol. 105 (1-2), 179–188. 10.1016/s0168-1656(03)00189-5 14511918

[B15] Boško R. Martiník J. Wawroszová S. Benešová K. Svoboda Z. Běláková S. (2024). Ergot alkaloids in rye flour marketed in Czech republic: comparison between ELISA and LC–MS methodologies. Food Anal. Methods 17, 787–794. 10.1007/s12161-024-02612-x

[B16] Brauer J. Wiechert R. Hahn A. Opatz T. (2024). Solving a mystery with classical and dual photoredox catalysis: application of nickel in the synthesis of ergot alkaloids. ChemRxiv. 10.26434/chemrxiv-2024-sss7l-v2 38728534

[B17] Bryła M. Szymczyk K. Jędrzejczak R. Roszko M. (2015). Application of liquid chromatography/ion trap mass spectrometry technique to determine ergot alkaloids in grain products. Food Technol. Biotechnol. 53 (1), 18–28. 10.17113/ftb.53.01.15.3770 27904328 PMC5068433

[B18] Carbonell-Rozas L. Gámiz-Gracia L. Lara F. J. García-Campaña A. M. (2021b). Determination of the main ergot alkaloids and their epimers in oat-based functional foods by ultra-high performance liquid chromatography tandem mass spectrometry. Molecules 26 (12), 3717. 10.3390/molecules26123717 34207051 PMC8234484

[B19] Carbonell-Rozas L. Hernández-Mesa M. Righetti L. Monteau F. Lara F. J. Gámiz-Gracia L. (2022). Ion mobility-mass spectrometry to extend analytical performance in the determination of ergot alkaloids in cereal samples. J. Chromatogr. A 1682, 463502. 10.1016/j.chroma.2022.463502 36174373

[B20] Carbonell-Rozas L. Mahdjoubi C. K. Arroyo-Manzanares N. García-Campaña A. M. Gámiz-Gracia L. (2021a). Occurrence of ergot alkaloids in barley and wheat from Algeria. Toxins 13 (5), 316. 10.3390/toxins13050316 33925104 PMC8145663

[B21] Chen J. J. Han M. Y. Gong T. Qiao Y. M. Yang J. L. Zhu P. (2019). Epigenetic modification enhances ergot alkaloid production of *Claviceps purpurea* . Biotechnol. Lett. 41, 1439–1449. 10.1007/s10529-019-02750-x 31659576

[B22] Cherewyk J. E. Blakley B. R. Al-Dissi A. N. (2024). The C-8-S-isomers of ergot alkaloids — a review of biological and analytical aspects. Mycotoxin Res. 40, 1–17. 10.1007/s12550-023-00507-0 37953416 PMC10834577

[B23] Cherewyk J. E. Parker S. E. Blakley B. R. Al-Dissi A. N. (2020). Assessment of the vasoactive effects of the (S)-epimers of ergot alkaloids *in vitro* . J. Anim. Sci. 98 (7), skaa203. 10.1093/jas/skaa203 32629472 PMC7373324

[B24] Chung S. W. C. (2021). A critical review of analytical methods for ergot alkaloids in cereals and feed and in particular suitability of method performance for regulatory monitoring and epimer-specific quantification. Food Addit. Contam. Part A Chem. Anal. Control Expo. Risk Assess. 38 (6), 997–1012. 10.1080/19440049.2021.1898679 33784227

[B25] Claßen J. Aupert F. Reardon K. F. Solle D. Scheper T. (2017). Spectroscopic sensors for in-line bioprocess monitoring in research and pharmaceutical industrial application. Anal. Bioanal. Chem. 409 (3), 651–666. 10.1007/s00216-016-0068-x 27900421

[B26] Crews C. (2015). Analysis of ergot alkaloids. Toxins (Basel). 7 (6), 2024–2050. 10.3390/toxins7062024 26046699 PMC4488688

[B27] Davis K. A. Jones A. M. Panaccione D. G. (2023). Two satellite gene clusters enhance ergot alkaloid biosynthesis capacity of *Aspergillus leporis* . Appl. Environ. Microbiol. 89, e0079323–23. 10.1128/aem.00793-23 37432119 PMC10467348

[B28] Davis K. A. Sampson J. K. Panaccione D. G. (2020). Genetic reprogramming of the ergot alkaloid pathway of *Metarhizium brunneum* . Appl. Environ. Microbiol. 86 (19), 012511–e1320. 10.1128/AEM.01251-20 PMC7499049 32769181

[B29] Doi Y. Wakana D. Takeda H. Tanaka E. Hosoe T. (2022). Production of ergot alkaloids by the Japanese isolate *Claviceps purpurea var. agropyri* on rice medium. Adv. Microbiol. 12 (4), 254–269. 10.4236/aim.2022.124019

[B30] Dousa M. Dubovská M. (2010). High-performance liquid chromatographic determination of dihydroergocristine in a pharmaceutical formulation with fluorescence detection. J. AOAC Int. 93 (1), 97–101. 10.1093/jaoac/93.1.97 20334170

[B31] Esmonde-White K. A. Cuellar M. Uerpmann C. Lenain B. Lewis I. R. (2017). Raman spectroscopy as a process analytical technology for pharmaceutical manufacturing and bioprocessing. Anal. Bioanal. Chem. 409 (3), 637–649. 10.1007/s00216-016-9824-1 27491299 PMC5233728

[B32] Faassen S. M. Hitzmann B. (2015). Fluorescence spectroscopy and chemometric modeling for bioprocess monitoring. Sensors (Basel) 15 (5), 10271–10291. 10.3390/s150510271 25942644 PMC4481931

[B33] Farid N. F. Abdelwahab N. S. (2019). Development and validation of different chromatographic methods for analysis of cabergoline in the presence of its degradation products: studying degradation profile. Chromatographia 82, 1555–1569. 10.1007/s10337-019-03763-4

[B34] Felici C. E. Wang C. C. Fernandez L. P. Gomez M. R. A. (2015). Simultaneous separation of ergot alkaloids by capillary electrophoresis after cloud point extraction from cereal samples. Electrophoresis 36 (2), 341–347. 10.1002/elps.201400215 25257749

[B35] Gillespie C. Wasalathanthri D. P. Ritz D. B. Zhou G. Davis K. A. Wucherpfennig T. (2022). Systematic assessment of process analytical technologies for biologics. Biotechnol. Bioeng. 119 (2), 423–434. 10.1002/bit.27990 34778948

[B36] Gross M. Curtui V. Usleber E. (2018). Detection of total ergot alkaloids in cereal flour and in bread by a generic enzyme immunoassay method. J. AOAC Int. 101 (3), 618–626. 10.5740/jaoacint.17-0346 28964275

[B37] Hasanpour F. Taei M. Banitaba S. H. Heidari M. (2017). Template synthesis of maghemite nanoparticle in carboxymethyl cellulose and its application for electrochemical cabergoline sensing. Mater Sci. Eng. C Mater Biol. Appl. 76, 88–93. 10.1016/j.msec.2017.02.128 28482603

[B38] Himmelsbach M. Ferdig M. Rohrer T. (2014). Analysis of paspalic acid, lysergic acid, and *iso*-lysergic acid by capillary zone electrophoresis with UV- and quadrupole time-of-flight mass spectrometric detection. Electrophoresis 35, 1329–1333. 10.1002/elps.201300224 24115177

[B39] Holderied I. Rychlik M. Elsinghorst P. W. (2019). Optimized analysis of ergot alkaloids in rye products by liquid chromatography-fluorescence detection applying lysergic acid diethylamide as an internal standard. Toxins 11, 184. 10.3390/toxins11040184 30925708 PMC6520901

[B40] Hu M. Zhou Y. Du S. Zhang X. Tang S. Yang Y. (2023). Construction of an efficient *Claviceps paspali* cell factory for lysergic acid production. Front. Bioeng. Biotechnol. 10, 1093402. 10.3389/fbioe.2022.1093402 36760750 PMC9905238

[B41] Isoko K. Cordiner J. L. Kis Z. Moghadam P. Z. (2024). Bioprocessing 4.0: a pragmatic review and future perspectives. Digit. Discov. 3, 1662–1681. 10.1039/D4DD00127C

[B42] Jastrzębski M. K. Kaczor A. A. Wróbel T. M. (2022). Methods of lysergic acid synthesis—the key ergot alkaloid. Molecules 27 (21), 7322. 10.3390/molecules27217322 36364148 PMC9654825

[B43] Jyothirmayee C. A. Raju K. N. (2014). RP-HPLC method for the estimation of cabergoline in pharmaceutical formulations. Int. J. Res. Pharm. Chem. 4 (4), 977–981.

[B44] Kodisch A. Oberforster M. Raditschnig A. Rodemann B. Tratwal A. Danielewicz J. (2020). Covariation of ergot severity and alkaloid content measured by HPLC and one ELISA method in inoculated winter rye across three isolates and three European countries. Toxins 12 (11), 676. 10.3390/toxins12110676 33114663 PMC7692364

[B45] Komarova E. L. Tolkachev O. N. (2001a). The chemistry of peptide ergot alkaloids. Part 1. Classification and chemistry of ergot peptides. Pharm. Chem. J. 35, 504–513. 10.1023/A:1014050926916

[B46] Komarova E. L. Tolkachev O. N. (2001b). The chemistry of peptide ergot alkaloids. part 2. analytical methods for determining ergot alkaloids. Pharm. Chem. J. 35, 542–549. 10.1023/A:1014706301632

[B47] Kotzagiorgis E. C. Michaleas S. Antoniadou-Vyza E. (2007). Improved photostability indicating ion-pair chromatography method for pergolide analysis in tablets and in the presence of cyclodextrins. J. Pharm. Biomed. Analysis 43 (4), 1370–1375. 10.1016/j.jpba.2006.11.019 17188445

[B48] Kozák L. Szilágyi Z. Vágó B. Kakuk A. Tóth L. Molnár I. (2018). Inactivation of the indole-diterpene biosynthetic gene cluster of *Claviceps paspali* by *Agrobacterium*-mediated gene replacement. Appl. Microbiol. Biotechnol. 102 (7), 3255–3266. 10.1007/s00253-018-8807-x 29457197 PMC5854541

[B49] Králová M. Frébortová J. Pěnčík A. Frébort I. (2021). Overexpression of Trp-related genes in *Claviceps purpurea* leading to increased ergot alkaloid production. New Biotechnol. 61, 69–79. 10.1016/j.nbt.2020.11.003 33188977

[B50] Kumar K. K. Nadh R. V. (2011). Development and validation of hplc method for the estimation of nicergoline in marketed formulations. Rasayan JChem 4 (4), 885–889.

[B51] Kuner M. Kühn S. Haase H. Meyer K. Koch M. (2021). Cleaving ergot alkaloids by hydrazinolysis — a promising approach for a sum parameter screening method. Toxins (Basel). 13 (5), 342. 10.3390/toxins13050342 34064772 PMC8151494

[B52] Lim L. R. Wong G. Go M. K. Yew W. S. (2023). Increasing lysergic acid levels for ergot alkaloid biosynthesis: directing catalysis via the F-G loop of Clavine oxidases. Front. Microbiol. 14, 1150937. 10.3389/fmicb.2023.1150937 37007471 PMC10060963

[B53] Liu H. Jia Y. (2017). Ergot alkaloids: synthetic approaches to lysergic acid and clavine alkaloids. Nat. Product. Rep. 34 (4), 411–432. 10.1039/c6np00110f 28300233

[B54] Ma Y. Yan J. Yang L. Yao Y. Wang L. Gao S. ‒S. (2022). A hybrid system for the overproduction of complex ergot alkaloid chanoclavine. Front. Bioeng. Biotechnol. 10, 1095464. 10.3389/fbioe.2022.1095464 36619381 PMC9811125

[B55] Meneghetti F. Ferraboschi P. Grisenti P. Reza Elahi S. Mori M. Ciceri S. (2020). Crystallographic and NMR investigation of ergometrine and methylergometrine, two alkaloids from *Claviceps purpurea* . Molecules 25 (2), 331. 10.3390/molecules25020331 31947568 PMC7024318

[B56] Mitra S. Murthy G. S. (2022). Bioreactor control systems in the biopharmaceutical industry: a critical perspective. Syst Microbiol Biomanuf 2, 91–112. 10.1007/s43393-021-00048-6 38624976 PMC8340809

[B57] Mohammadi S. Z. Beitollahi H. Allahabadi H. Rohani T. (2019). Disposable electrochemical sensor based on modified screen printed electrode for sensitive cabergoline quantification. J. Electroanal. Chem. 847, 113223. 10.1016/j.jelechem.2019.113223

[B58] Nielsen C. A. Folly C. Hatsch A. Molt A. Schröder H. O'Connor S. E. (2014). The important ergot alkaloid intermediate chanoclavine-I produced in the yeast *Saccharomyces cerevisiae* by the combined action of *EasC* and *EasE* from *Aspergillus japonicas* . Microb. Cell Fact. 13, 95. 10.1186/s12934-014-0095-2 25112180 PMC4249865

[B59] Oellig C. (2017). Lysergic acid amide as chemical marker for the total ergot alkaloids in rye flour - determination by high-performance thin-layer chromatography-fluorescence detection. J. Chromatogr. A 1507, 124–131. 10.1016/j.chroma.2017.05.043 28554863

[B60] Oellig C. Melde T. (2016). Screening for total ergot alkaloids in rye flour by planar solid phase extraction–fluorescence detection and mass spectrometry. J. Chromatogr. A 1441, 126–133. 10.1016/j.chroma.2016.02.075 26947163

[B61] Önal A. Sağırlı O. Şensoy D. (2007). Selective LC determination of cabergoline in the bulk drug and in tablets: *in vitro* dissolution studies. Chromatographia 65, 561–567. 10.1365/s10337-007-0211-0

[B62] Pankin D. Povolotckaia A. Borisov E. Povolotskiy A. Borzenko S. Gulyaev A. (2023). Investigation of spectroscopic peculiarities of ergot-infected winter wheat grains. Foods 12 (18), 3426. 10.3390/foods12183426 37761134 PMC10528831

[B63] Poapolathep S. Klangkaew N. Zhang Z. Giorgi M. Logrieco A. F. Poapolathep A. (2021). Simultaneous determination of ergot alkaloids in swine and dairy feeds using ultra high-performance liquid chromatography-tandem mass spectrometry. Toxins 13, 724. 10.3390/toxins13100724 34679017 PMC8540808

[B64] Pukngam P. Burana-osot J. (2013). Development and validation of a stability-indicating HPLC method for determination of bromocriptine mesylate in bulk drug and tablets. Curr. Pharm. Anal. 9 (1), 92–101. 10.2174/157341213804806106

[B65] Qiao Y.-M. Wen Y.-H. Gong T. Chen J.-J. Chen T.-J. Yang J.-L. (2022). Improving ergometrine production by *easO* and *easP* knockout in *Claviceps paspali* . Fermentation 8 (6), 263. 10.3390/fermentation8060263

[B66] Radi A. El-Shahawi M. S. Elmogy T. (2005). Differential pulse voltammetric determination of the dopaminergic agonist bromocriptine at glassy carbon electrode. J. Pharm. Biomed. Anal. 37 (1), 195–198. 10.1016/j.jpba.2004.10.013 15664762

[B67] Rambabu C. Jyothirmayee C. A. Raju K. N. (2012). Spectrophotometric determination of cabergoline in tablet dosage form. Int. J. Pharm. Chem. Biol. Sci. 2 (1), 72–77.

[B68] Rathore A. S. Mishra S. Nikita S. Priyanka P. (2021). Bioprocess control: current progress and future perspectives. Life (Basel) 11 (6), 557. 10.3390/life11060557 34199245 PMC8231968

[B69] Rizk M. Mahmoud Z. M. Azab M. M. (2022). Spectrofluorimetric and stability-indicating thin layer chromatographic methods for determination of cabergoline, a prolactin inhibitor in pharmaceuticals. Spectrochim. Acta A Mol. Biomol. Spectrosc. 281, 121639. 10.1016/j.saa.2022.121639 35872427

[B70] Schiff P. L. (2006). Ergot and its alkaloids. Am. J. Pharm. Educ. 70 (5), 98. 10.5688/aj700598 17149427 PMC1637017

[B71] Schlembach I. Grünberger A. Rosenbaum M. A. Regestein L. (2021). Measurement techniques to resolve and control population dynamics of mixed-culture processes. Trends Biotechnol. 39 (10), 1093–1109. 10.1016/j.tibtech.2021.01.006 33573846 PMC7612867

[B72] Schummer C. Zandonella I. van Nieuwenhuyse A. Moris G. (2020). Epimerization of ergot alkaloids in feed. Heliyon 6 (6), e04336. 10.1016/j.heliyon.2020.e04336 32637710 PMC7330613

[B73] Shahid M. G. Nadeem M. Gulzar A. Saleem M. Rehman H. U. Ghafoor G. Z. (2020). Novel ergot alkaloids production from *Рenicillium citrinum* employing response surface methodology technique. Toxins (Basel). 12 (7), 427. 10.3390/toxins12070427 PMC7405006 32610508

[B74] Shank B. R. Ofner C. M. (2010). Multitemperature stability and degradation characteristics of pergolide mesylate oral liquid. J. Pharm. Pract. 23 (6), 570–574. 10.1177/0897190010375851 21507864

[B75] Sheshegova T. K. Shchekleina L. M. Antipova T. V. Zhelifonova V. P. Kozlovskiy A. G. (2021). Search for rye and wheat genotypes which are resistant to *Claviceps Purpurea* Fr. Tul. hamper accumulation ergoalkaloids sclerotia. Sel’skokhoz. Boil. [Agric. Biol.]. 56 (3), 549–558. 10.15389/agrobiology.2021.3.549rus

[B76] Sheshegova T. K. Shchekleina L. M. Zhelifonova V. P. Antipova T. V. Baskunov B. P. Kozlovsky A. G. (2019). A resistance of rye varieties to ergot and ergot alkaloid content in *Claviceps purpurea* sclerotia on the Kirov region environments. Mikol. i Fitopatol. 53 (3), 177–182. 10.1134/S0026364819030127

[B77] Silva Â. Mateus A. R. S. Barros S. C. Silva A. S. (2023). Ergot alkaloids on cereals and seeds: analytical methods, occurrence, and future perspectives. Molecules 28 (20), 7233. 10.3390/molecules28207233 37894711 PMC10609535

[B78] Soraya H. Jaut V. Schneider R. J. (2023). Ergometrine sensing in rye flour by a magnetic bead-based immunoassay followed by flow injection analysis with amperometric detection. Talanta 254, 124172. 10.1016/j.talanta.2022.124172 36535211

[B79] Sultan M. A. Maher H. M. Alzoman N. Z. Alshehri M. M. Rizk M. S. Elshahed M. S. (2013). Capillary electrophoretic determination of antimigraine formulations containing caffeine, ergotamine, paracetamol and domperidone or metoclopramide. J. Chromatogr. Sci. 51, 502–510. 10.1093/chromsci/bms175 23180758

[B80] Veršilovskis A. Mulder P. P. J. Pereboom-de Fauw D. P. K. H. de Stoppelaar J. de Nijs M. (2020). Simultaneous quantification of ergot and tropane alkaloids in bread in The Netherlands by LC-MS/MS. Food Addit. Contam. Part B Surveill. 13 (3), 215–223. 10.1080/19393210.2020.1771777 32482157

[B81] Volnin A. A. Savin P. S. (2022). Ergot *Claviceps purpurea* (Fries) Tulasne alkaloid diversity and virulence: evolution, genetic diversification, and metabolic engineering (review). Sel’skokhoz. Boil. Agric. Biol. . 57 (5), 852–881. 10.15389/agrobiology.2022.5.852rus

[B82] Volnin A. A. Tsybulko N. S. Savin P. S. Myasnikova S. B. (2022). Express-methods for selective and nonselective determination of indole alkaloids. Veterinary Med. Zootechnics Biotechnol. 1 (12), 117–124. 10.36871/vet.zoo.bio.202212115

[B83] Volnin A. A. Tsybulko N. S. Savin P. S. Myasnikova S. B. Bobyleva R. J. (2023). Biocollection of pharmaceutical parasitic strains of *Claviceps purpurea* - base for selection of new lines producing ergoalkaloids *in vitro* . Problems Biol. Med. Pharm. Chem. 26 (8), 22–31. 10.29296/25877313-2023-08-03

[B84] Wong G. Lim L. R. Tan Y. Q. Go M. K. Bell D. J. Freemont P. S. (2022). Reconstituting the complete biosynthesis of D-lysergic acid in yeast. Nat. Commun. 13 (1), 712. 10.1038/s41467-022-28386-6 35132076 PMC8821704

[B85] Yao Y. Wang W. Shi W. Yan R. Zhang J. Wei G. (2022). Overproduction of medicinal ergot alkaloids based on a fungal platform. Metab. Eng. 69, 198–208. 10.1016/j.ymben.2021.12.002 34902590

[B86] Zvonkova E. N. Lapa G. B. Glotov A. A. Anufrieva V. V. Monakhova T. E. Sheichenko V. I. (2000). Epimerization of 2-bromergocryptines. Pharm. Chem. J. 34, 259–260. 10.1007/BF02524635

